# Bayesian belief network model to predict human-wildlife conflict in protected areas

**DOI:** 10.1038/s41598-026-56081-9

**Published:** 2026-06-19

**Authors:** Santiago García-Lloré, Richard C. Stedman, Angela K. Fuller

**Affiliations:** 1 New York Cooperative Fish and Wildlife Research Unit, Ashley School of Global Development and the Environment, Cornell University, Ithaca, NY 14853 USA; 2 Ashley School of Global Development and the Environment, Cornell University, Ithaca, NY 14853 USA; 3 U.S. Geological Survey, New York Cooperative Fish and Wildlife Research Unit, Ashley School of Global Development and the Environment, Cornell University, Ithaca, New York 14853 USA

**Keywords:** Human-wildlife conflict, Bayesian belief network, Protected areas, Governance, Wildlife acceptance, Habitat quality, Environmental social sciences, Conservation biology

## Abstract

Human-wildlife conflict (HWC) poses a pervasive global challenge, affecting livelihoods and threatening biodiversity. To better anticipate and mitigate HWC risk, we developed a large-scale predictive model using a Bayesian Belief Network (BBN). We surveyed 1,011 park rangers across 135 terrestrial protected areas in three Andean countries, documenting recent HWC incidents involving wildlife persecution or killing, livestock depredation, crop damage, or threats to human safety and property. We identified key drivers of HWC risk, including governance, wildlife acceptance, participation, and habitat quality. A sensitivity analysis revealed that enhancing governance and improving wildlife acceptance could reduce HWC risk by > 85%. The BBN model demonstrated scalability, effectively identifying strategies to reduce HWC risk at multiple scales, from individual protected areas to national networks. Our findings highlight the importance of strengthening governance, increasing wildlife acceptance, and enhancing community participation in conservation efforts. BBNs provide a flexible, cost-effective, and data-driven tool to guide protected areas and wildlife managers in monitoring, anticipating, and making informed decisions to mitigate conflict and promote coexistence.

**Supplementary Information:**

The online version contains supplementary material available at 10.1038/s41598-026-56081-9.

## Introduction

Human-wildlife conflict (HWC) emerges from the negative interactions between people and wildlife that cause harm to one or both parties^1,2^. These conflicts are inherently complex, characterized by the lack of optimal solutions, scientific uncertainties, and conflicting perceptions of values and attitudes among stakeholders^3,4^. HWC has diverse underlying causes, including social, economic, and environmental dimensions^5^. The conflict between people and wildlife often disrupts livelihoods through crop-raiding and livestock attacks^6^ and damage to domestic animals^7^. In marine ecosystems, though less researched, conflicts also occur, including damage to fishing gear and competition-induced conflicts in capture fishing. Furthermore, HWC can threaten human health through disease transmission^8,9^, and in some cases, though direct attacks on people^10,11^. These conflicts not only affect human well-being, but also contribute to biodiversity loss, often through retaliatory or preventative killing (as well as injury or removal) of wildlife following conflict events, which can disproportionately impact large carnivores because of their low reproductive rates and frequent persecution^12,13^. Currently, 64% of large carnivores are at risk of extinction, 80% are experiencing population declines, and 80% have experienced range contractions.

Addressing the biodiversity crisis is imperative^14,15^, and the establishment of protected areas (PAs) has been one of the most widely accepted strategies for conserving biodiversity^16^. Created over 150 years ago, PAs have served as a refuge for wildlife^17^ and have played a crucial role in mitigating biodiversity loss and climate change impacts^18^. PAs host 87.6% of species listed as vulnerable, endangered or critically endangered, and cover 15.3% of the land and freshwater environments^19^. They can also contribute to human well-being^20–22^. Despite the benefits of PAs, studies indicate that they are insufficient to protect wildlife and are not exempt from HWC^23^. The conservation effectiveness of PAs for large carnivores has been questioned due to their biological traits such as large body sizes, large distribution ranges, and low population densities^24^. For example, a study in Europe demonstrated that while PAs can protect small wildlife species, in the case of large carnivores (*Lynx lynx*, *Canis lupus*, and *Ursus arctos*), PAs serve more as important stepping stones within broader landscapes rather than a solution for conservation^25^.

Several interacting factors reduce the effectiveness of PAs in mitigating HWC: (a) inadequate governance, indicating the lack or poor capacity of PAs to formulate policies, norms, and coordinate actions among stakeholders^26,27^; (b) degraded habitat quality due to landscape disturbances that are a product of human activities like farming, ranching, hunting, and infrastructure development, these disturbances affect functional and structural landscape connectivity, intensifying the competition between wildlife and humans for resources and spaces, while increasing the chance for negative human-wildlife interactions^28,29^; and (c) low acceptance of wildlife, due to actual or perceived damage resulting from negative interactions. Wildlife acceptance varies among wildlife species, influenced by cultural perceptions and attitudes^13,30^. The causes of wildlife decline within PAs are not mutually exclusive, and often, multiple causes operate simultaneously, exacerbating the challenges of managing wildlife within PAs.

The mitigation of HWC poses significant challenges due to the stochastic nature of both human and wildlife systems^31–33^. Current approaches to alleviate conflict have primarily focused on segregating wildlife from human territories, employing strategies such as fencing or designing areas intended exclusively for wildlife, like protected areas. A critical obstacle in addressing HWC is the absence of standardized, scalable methods to synthesize information across scales and simultaneously address the diverse drivers of conflict^10,34^, a need we infer by synthesizing prior recommendations to collect and assess key ecological and social information across contexts. The lack of a comprehensive tool capable of analyzing the multifaceted causes of HWC simultaneously hinders the development of holistic solutions.

One tool potentially useful for evaluating HWC risk is a Bayesian belief network (BBN), also known as a probability network. BBNs are multivariate models used to analyze relationships and patterns among multiple variables (qualitative and quantitative). They rely on Bayesian probability, allowing for probabilistic inference based on both prior knowledge and observed data^35,36^. One of BBN’s primary functions is to update probabilities as new evidence is introduced, making them particularly useful for problems with uncertainty or incomplete information. A BBN consists of a network of connected, discretized variables that represent probabilistic dependencies. These networks use prior information and observed data to infer the likelihood of different outcomes.

The BBN uses a directed acyclic graph (DAG), where nodes represent variables and edges define causal relationships or dependencies. The strength of these relationships is quantified through conditional probability tables (CPTs)^35,37^. This probabilistic framework allows BBNs to estimate event probabilities dynamically as new evidence becomes available. BBNs have been applied in various fields, including deforestation prediction in the Amazon rainforest^37^, road accident prediction^38^, classification of lead levels in drinking water systems^39^, ecosystem service trade-off analysis^40^, and ecological risk assessment^41^. The inherent characteristics of BBNs lend themselves to addressing complex dynamics such as those that exist in human-wildlife coexistence.

The primary objective of this research is to showcase the effectiveness of the BBNs approach in addressing HWC. Specifically, we aim to estimate the risk of future HWC occurrence while demonstrating the versatility of this approach to operate at various scales under conditions of high uncertainty. We posit that the PAs, including their edge zones, serve as hotspots of heightened interactions between wildlife and human settlements. We hypothesized that PAs with high habitat quality, strong governance, and high wildlife acceptance would exhibit a lower risk of HWC occurrence. Conversely, we anticipated that PAs with low habitat quality, weak governance, and low acceptance of wildlife would demonstrate a higher risk of HWC occurrence.

## Results

Between September 2020 and November 2021, we received 1417 responses from PAs employees (managers, park rangers, and other staff members);137 responses were excluded following review, including 96 that were categorized as NA due to not completing minimal information in the survey like PA name, and 41 that were removed because they represented duplicate responses. Although we included 194 PAs in the survey, we only received responses from 176. We did not receive responses from four PAs in Colombia, nine PAs in Ecuador, and nine PAs in Peru.

After reviewing and cleaning the survey data, 1,280 valid responses were included from 176 PAs of Colombia, Ecuador, and Peru (Supplementary Table 1). Respondents included 95 PA managers, 293 PA specialists, 893 park rangers, 9 PA assistants, and 61 PA staff in “other” categories among the three countries (Supplementary Table 2).

### BBN model results

We focus the BBN model on terrestrial PAs of Colombia, Ecuador, and Peru. Reflecting the multidisciplinary challenges of HWC described earlier, we used 15 variables in our BBN to predict the risk of HWC occurrence in the terrestrial protected areas of the three countries. Information on 11 variables derived from our online survey included participation, law enforcement, law acceptance, frequency of frequency of illegal wildlife crimes (hunting and fishing), distance between HWC events and human settlements, tourism as number of visitors, number of rangers in the PA, tolerance toward wildlife, attitude toward wildlife, education, and the frequency of reported activities against wildlife (Table 1). The means and standard deviations for each of these variables appear in the bottom box of each variable in the model figures.

**Table 1:** Descriptive statistics of the Bayesian Belief Network (BBN) variables from terrestrial protected areas in Colombia (*n* = 42), Ecuador (*n* = 35), and Peru (*n* = 58) were used to estimate the risk of human wildlife conflict (HWC) occurrence by each country at the protected area system level.

Variables	Description	Countries	# Protected areas	# Total responses (*N*)	# Valid responses (*n*)	Cases # No Answer	Mean	SD	Category count Min–Max	Categorical states for each variable
Participation	How often people try to influence decisions about forest and wildlife use in the protected area.	Colombia	42	175	150	25	3.6	1.4	1–5	Weekly Monthly Every six months Annually Never
		Ecuador	35	480	463	17	3.2	1.3		
		Peru	58	355	321	34	2.9	1.0		
Law enforcement	How hard it is to make sure people follow the rules in the protected area.	Colombia	42	175	150	25	2.0	0.6	1–3	Not difficult Slightly difficult Very difficult
		Ecuador	35	480	463	17	2.3	0.6		
		Peru	58	355	321	34	1.9	0.6		
Law acceptance	How often protected area rules are questioned or resisted by the local community.	Colombia	42	175	150	25	3.9	1.2	1–5	Weekly Monthly Every six months Annually Never
		Ecuador	35	480	463	17	3.3	1.3		
		Peru	58	355	320	35	4.0	1.1		
Wildlife Crime Frequency	How often did you receive complaints or were you alerted by members of the community about incidents of illegal hunting or fishing carried out by other members of the community within the protected area and its surrounding buffer zone?	Colombia	42	175	149	26	2.87	1.73	1–5	Weekly Monthly Every six months Annually Never
		Ecuador	35	480	460	21	3.3	1.4		
		Peru	58	355	304	51	3.4	1.5		
Distance	How far from human settlements human–wildlife conflicts are typically observed.	Colombia	42	175	92	83	1.4	0.5	1–2	Far Near
		Ecuador	35	480	227	253	1.2	0.4		
		Peru	58	355	108	247	1.2	0.4		
Tourism	The number of tourists visiting the protected area.	Colombia	42	175	168	7	2.0	0.7	1–3	Decrease Same Increase
		Ecuador	35	480	480	0	2.3	0.8		
		Peru	58	355	341	14	1.9	0.8		
Ranger	The number of park rangers working in the protected area	Colombia	42	175	168	7	2.3	0.7	1–3	Decrease Same Increase
		Ecuador	35	480	480	0	1.4	0.6		
		Peru	58	355	341	14	1.9	0.6		
Tolerance	The extent to which people agree that most damage caused by wildlife should be accepted without retaliation.	Colombia	42	175	168	7	2.7	1.3	1–5	Strongly disagree Disagree Neutral Agree Strongly agree
		Ecuador	35	480	479	1	2.5	1.3		
		Peru	58	355	335	20	2.8	1.3		
Attitude	How people feel about wildlife based on their experiences.	Colombia	42	175	150	25	2.2	0.8	1–3	Negative Neutral Positive
		Ecuador	35	480	463	17	2.0	0.7		
		Peru	58	355	320	25	2.2	0.7		
Education	How often protected area staff share information with communities about nature’s benefits and protected area rules.	Colombia	42	175	150	25	2.9	1.1	1–5	Weekly Monthly Every six months Annually Never
		Ecuador	35	480	463	17	2.7	1.0		
		Peru	58	355	355	0	2.9	1.0		
Human–wildlife conflict report frequency	How often people complain about wildlife problems, showing what is seen as acceptable or not in their community.	Colombia	42	175	92	83	2.6	1.0	1–5	Weekly Monthly Every six months Annually Never
		Ecuador	35	480	254	226	2.6	0.9		
		Peru	58	355	108	247	2.4	1.0		

We assigned numerical values for each categorical state variable to estimate a weighted mean (central tendency) for each variable, and the standard deviation (SD) was computed to understand the variability of the assigned values.

**Fig. 1:**
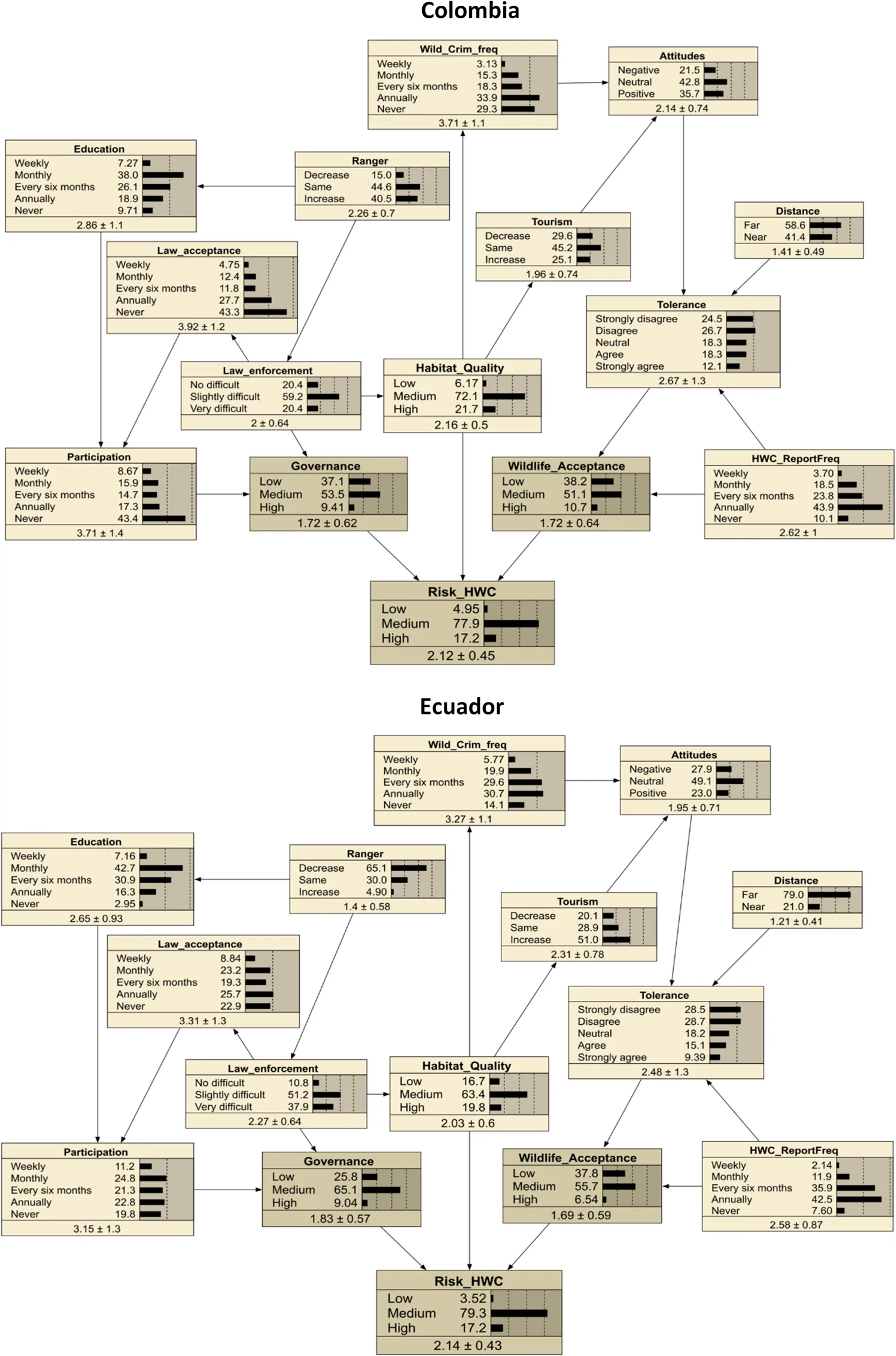
Bayesian Network Model predicting risk of human–wildlife conflict (HWC) in terrestrial protected areas of Colombia (*n* = 42) and Ecuador (*n* = 35). Wild_Crim_freq = wildlife crime frequency, HWC_ReportFreq = human-wildlife conflict report frequency, Risk_HWC = risk of human-wildlife conflict.

**Fig. 2:**
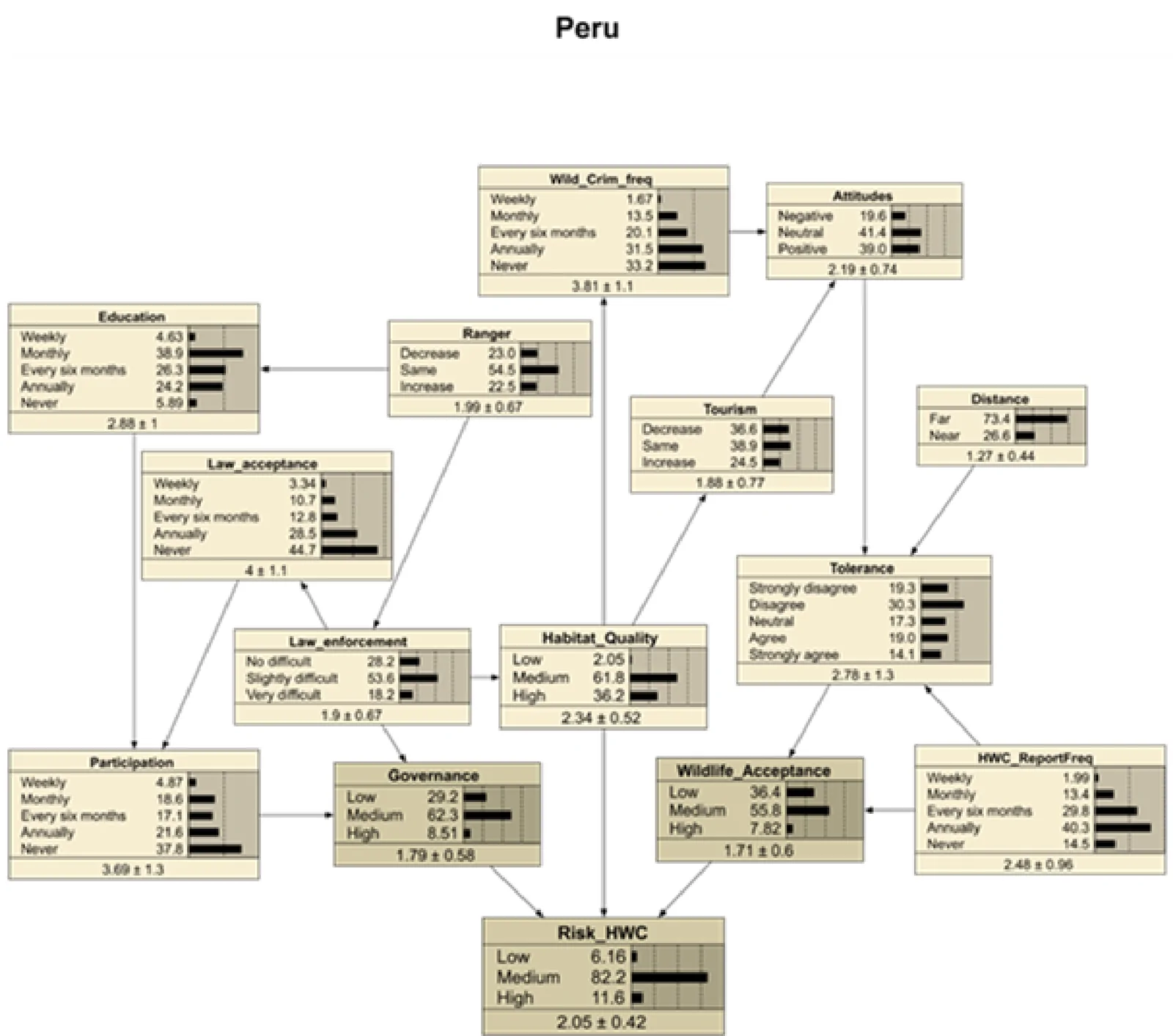
Bayesian Network Model predicting risk of human–wildlife conflict (HWC) in terrestrial protected areas of Peru (*n* = 58). Wild_Crim_freq = wildlife crime frequency, HWC_ReportFreq = human-wildlife conflict report frequency, Risk_HWC = risk of human-wildlife conflict.

We ran separate models for each country to preserve country-specific dynamics in protected area systems that differ substantially in the number of protected areas, ecosystem types, staffing capacity, management structures, governance arrangements, and financial resources. Modeling countries separately reduced the risk of masking important contextual differences and helped generate results that are more interpretable, policy-relevant, and practical for decision-makers in each national context. We interpret greater potential for coexistence between people and wildlife if the model results in a higher percentage of low and medium risk of HWC than of high-risk HWC. In contrast, if the high risk of HWC surpasses the low and medium risks, this suggests potential conflicts. Figure 1 shows the results of the model for terrestrial protected areas in Colombia and Ecuador, and Fig. 2 shows results in Peru. In Colombia, the proportion of high risk of HWC (17.2%) was nearly four times greater than the low risk (4.95%), with low governance (37.1%) also dominating over high governance (9.41%). In Ecuador, the high risk of HWC (17.2%) was almost five times higher than the low risk (3.5%), while the medium risk prevailed (79.2%); here, low governance (see Table 2 for variable definitions and operationalization) (25.8%) exceeded high governance (9.4%) by a factor of 2.5. In Peru, the difference between high (11.6%) and low (6.2%) risks was almost the double, and the medium risk was dominant (82.2%). Similarly, low governance (29.2%) was more than 3.0 times higher than high governance (8.5%). Figure 1.

### Sensitivity analysis

We conducted a one-way sensitivity analysis to identify the factors that had the greatest influence (variance reduction) on the outcome of interest, the risk of HWC. We performed the sensitivity analysis by systematically varying the values of one variable at a time to determine the effects on the risk of HWC. We then evaluated the sensitivity of the risk of HWC using the variable with the highest sensitivity. We used the software Netica 6.07 for all calculations.

**Fig. 3:**
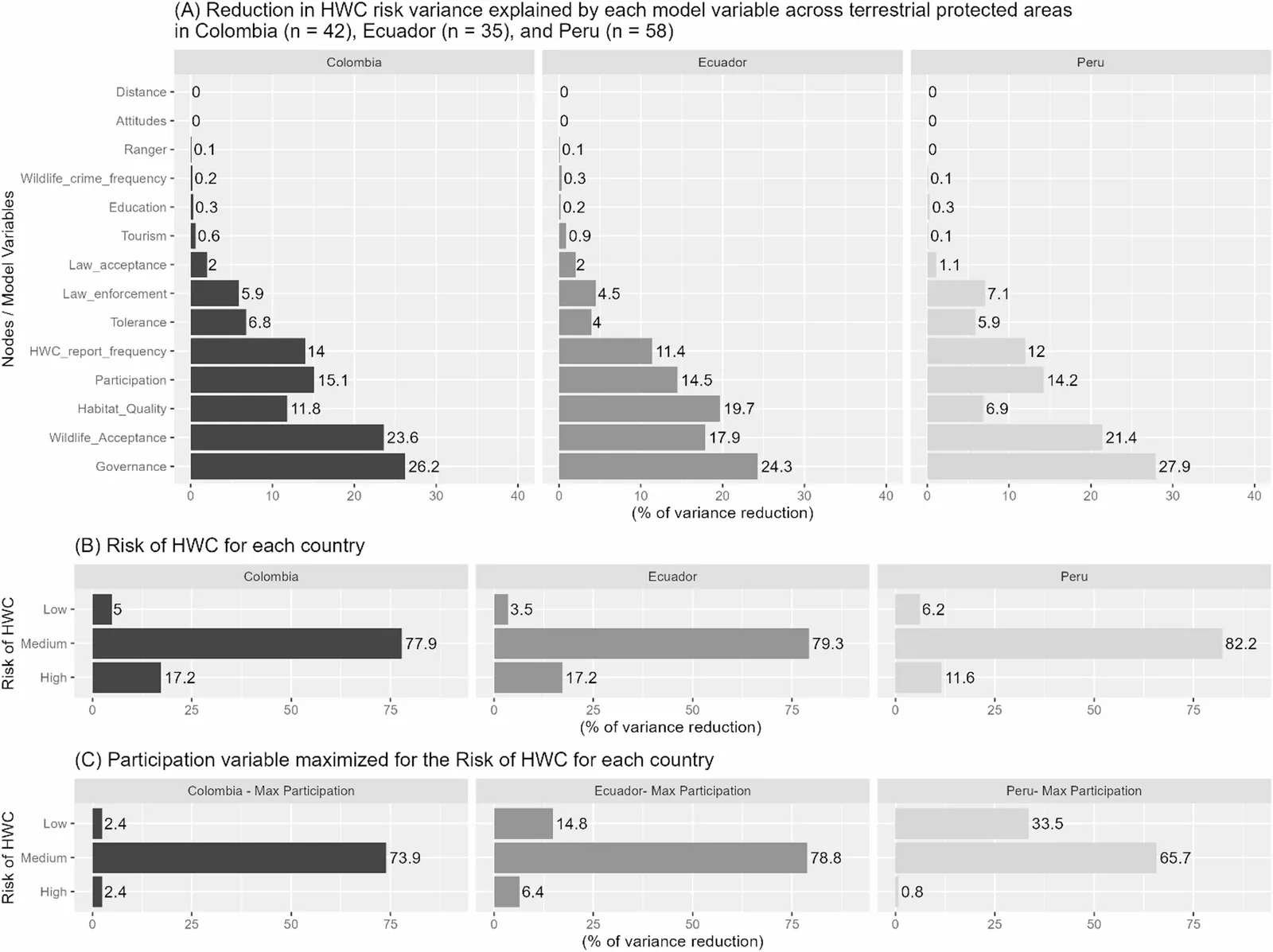
Sensitivity analysis of the probability of human–wildlife conflict (HWC) risk in terrestrial protected areas of Colombia, Ecuador, and Peru. Panels show (**A**) variance reduction by variable, (**B**) baseline probabilities of HWC risk, and (**C**) changes in high-risk levels after maximizing participation.

In Colombia, the variables with the largest impact on reducing the variance in the Risk of HWC in PAs were Governance (26.2%), Wildlife Acceptance (23.6%), and Participation (15.1%). In Ecuador, these variables were Governance (24.3%), Habitat Quality (19.7%), and Wildlife Acceptance (17.9%). Finally, in Peru, the top three variables were Governance (27.9%), Wildlife Acceptance (21.4%), and Participation (14.2%). We maximized the participation variable to observe its impact on the Risk of HWC for each country. After optimizing participation, we observed a substantial decrease in the high risk of HWC, with reductions of 86%, 62.8%, and 93.1% in the terrestrial protected areas of Colombia, Ecuador, and Peru, respectively. Figure 3.

**Fig. 4:**
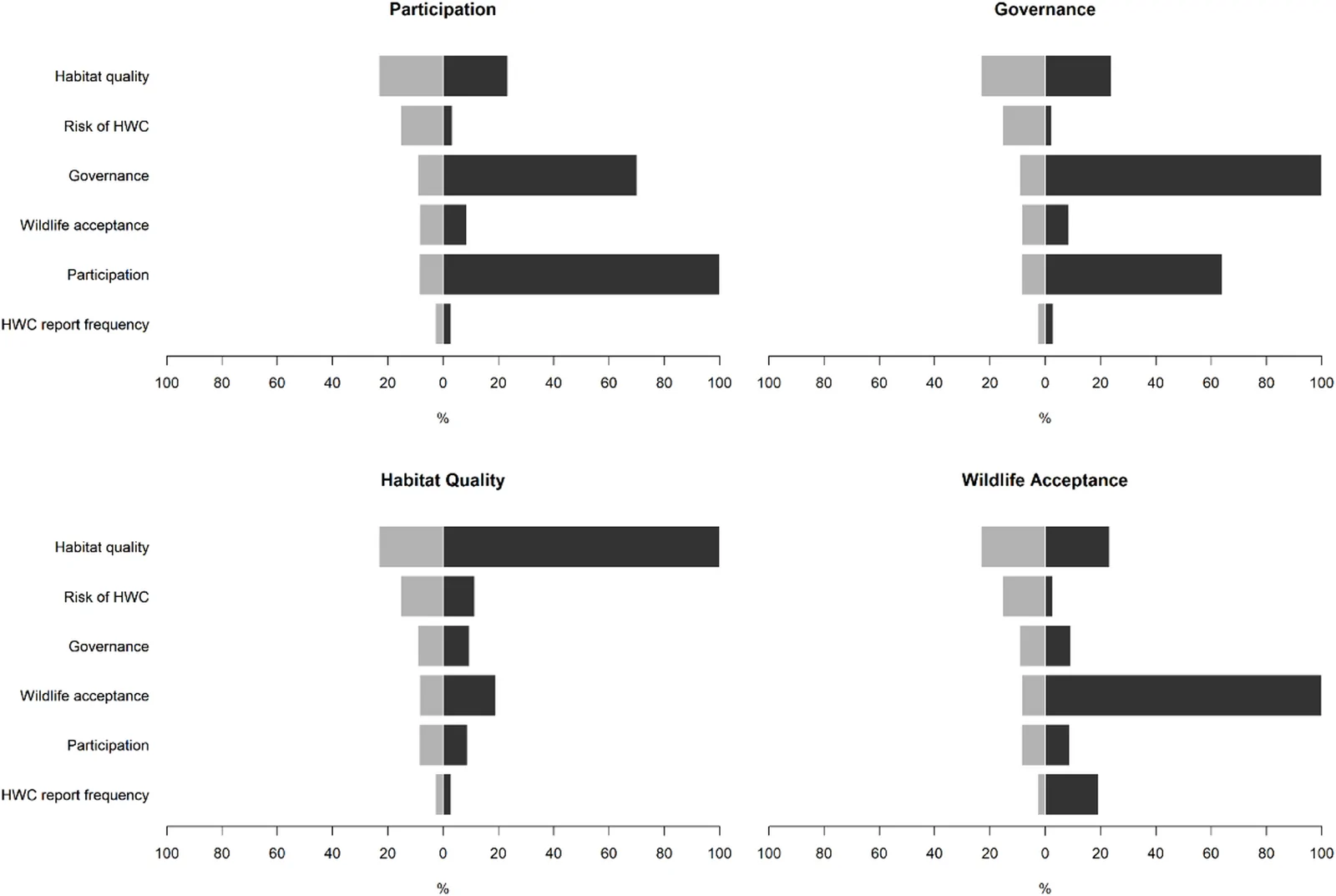
Average sensitivity analysis of the risk of human–wildlife conflict (HWC) in terrestrial protected areas across Colombia, Ecuador, and Peru. The Y-axis shows the top variables with the greatest variance reduction, with split bars representing original.

We estimated the average variance reduction in the risk of HWC by maximizing governance, participation, habitat quality, and wildlife acceptance. The sensitivity analysis showed that maximizing governance produced the highest reduction, lowering the risk from 15.3% to 2.3%. Participation also had a strong effect, reducing the risk from 15.3% to 3.2%. Maximizing habitat quality decreased the risk to 11.28%, while wildlife acceptance reduced it to 2.7%. Figure 4.

### Model scalability

Examining HWC across scales is essential for tailored interventions, ensuring efficient resource allocation, and assessing ecological impacts. Different conflicts demand diverse approaches, from local community engagement to broader regional or national strategies. Understanding conflicts at various scales enables a holistic perspective that promotes effective coexistence between humans and wildlife.

To showcase our model’s scalability, we assessed the risk of HWC at the protected area level by running the BBN in one PA per country: the Sanctuary of Plantas Medicinales in Colombia, Yasuní National Park in Ecuador, and the Historic Sanctuary of Machu Picchu in Peru. In Plantas Medicinales, governance was the most influential factor, reducing the variance in HWC by 26.3%, followed by wildlife acceptance (23.1%) and Participation (14.8%). In Yasuní, governance was the strongest driver, reducing variance by 24.1%, with habitat quality (18.9%) and wildlife acceptance (18.3%) also playing key roles. In Machu Picchu, governance again had the greatest effect (27.9%), followed by wildlife acceptance (21.1%) and participation (14.1%).

**Fig. 5:**
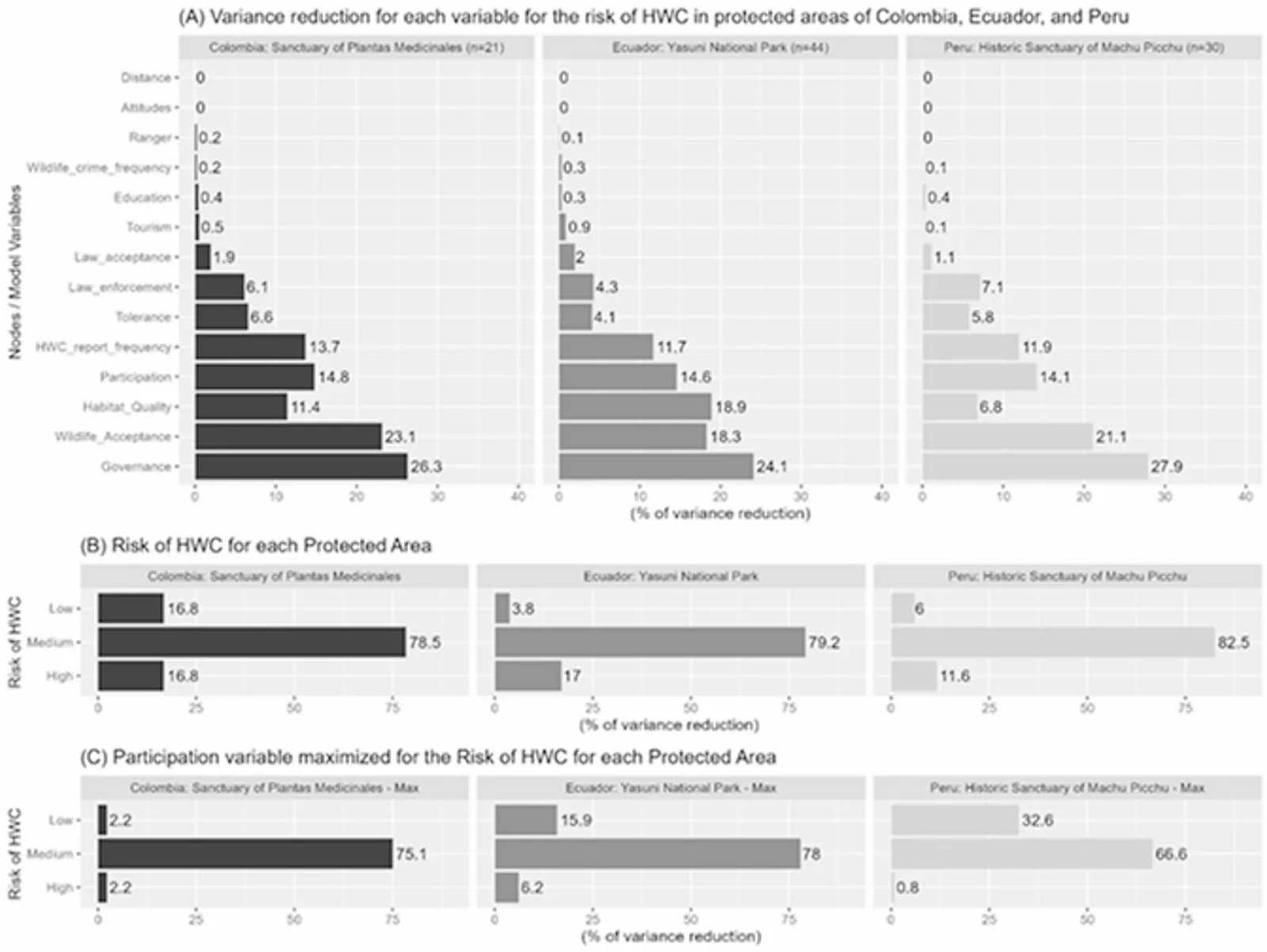
Sensitivity analysis of human–wildlife conflict (HWC) risk at the protected area level for the Sanctuary of Plantas Medicinales (Colombia), Yasuní National Park (Ecuador), and the Historic Sanctuary of Machu Picchu (Peru). Panels show (**A**) variance reduction by variable, (**B**) baseline probabilities of high-risk HWC, and (**C**) changes in high-risk HWC after maximizing participation.

The baseline probability of high-risk HWC was 16.8% in Plantas Medicinales, 17% in Yasuní, and 11.6% in Machu Picchu. When participation was maximized, the high-risk category dropped substantially, with reductions of 86.9%, 63.5%, and 93.1% in Plantas Medicinales, Yasuní, and Machu Picchu, respectively. Figure 5.

## Discussion

Our research leveraged the power of Bayesian Belief Networks, focusing on terrestrial PAs of Colombia, Ecuador, and Peru, and revealed that governance, wildlife acceptance, and participation were the most influential factors in decreasing the risk of HWC. The BBN model is a flexible, highly adaptable, and reliable tool that functions at multiple scales. The BBN models integrated the expert opinion of PA managers and staff, data on HWC events, and GIS-derived information on habitat quality within protected areas to graphically represent the dynamics of HWC across PA systems at a country level. This approach is well suited for problems with high uncertainty and a lack of coordination among managers. It provides a probabilistic framework to model and navigate uncertain scenarios, presents a clear graphical representation of interdependencies, and facilitates decentralized decision-making due to its capacity to analyze HWC at different scales.

We recognize important limitations in the data, especially regarding the social variables derived from manager surveys, which constitute proxy measures based on managerial perceptions rather than direct measurements of community behavior or lived experiences. Several nodes reflect protected area staff and ranger perspectives (e.g., ranger perceptions of community attitudes toward wildlife, perceived complaint frequency, and outreach efforts) and therefore capture staff awareness, institutional practices, and reporting dynamics as much as community conditions. In this context, we included two conceptually distinct variables to better represent different dimensions of human–wildlife interactions and reporting processes. The Human–wildlife conflict report frequency (HWC_report_freq) variable captures how often protected area staff receive complaints about HWC incidents within the protected area and its 3 km buffer and is interpreted as a proxy for both exposure to conflict and the likelihood that such events are formally reported through institutional channels. This reflects not only the occurrence of conflicts, but also reporting behavior, trust in authorities, and the degree of engagement between communities and protected area management. In contrast, the Wildlife crime frequency (Wild_crim_freq) variable measures the frequency with which staff are informed about incidents of illegal hunting or fishing, capturing a different behavioral dimension related to community responses to harmful activities toward wildlife. Higher reporting of wildlife crime is interpreted as indicative of increased awareness, lower tolerance for illegal activities, and potentially stronger pro-conservation attitudes or social pressures within communities. Although both variables rely on reported information and may be influenced by institutional capacity, monitoring effort, and reporting biases, they were retained because they reflect complementary—rather than redundant—processes: one related to conflict reporting and human–wildlife interactions, and the other to community responses to wildlife crime and norm enforcement. To evaluate whether both variables were measuring the same dimension, we conducted Spearman rank correlation analyses. Results indicated low-to-moderate associations between HWC_report_freq and Wild_crim_freq across the full sample and within countries (Supplementary Table S3).

Together, these proxies help capture variation in reporting culture and community-wildlife dynamics that are relevant for understanding patterns of human–wildlife conflict within protected areas.

Good governance is critical for preventing HWC or reducing its impacts as it establishes effective policies, promotes community engagement, and fosters sustainable management practices. That said, weak governance was 2.8 times more prevalent than strong governance across PAs in the three countries. If strong governance were to be maximized, the risk of HWC decreases from 15.33% to 2.3%. This dramatic reduction in HWC supports our hypothesis regarding the negative relationship between strong governance and the risk of HWC events in PAs. The differences between weak and strong governance among the three countries can be explained by the centralized governance model, where the state is responsible for overseeing national policies, environmental regulations, and stakeholder engagement within protected areas. This top-down approach limits the capacity of PAs to achieve some of the principles of good governance like inclusivity, fairness, transparency, accountability, legitimacy, direction, performance, and capability^42^ that are essential for effective management of natural resources. Certainly, weak governance increases HWC risks by allowing the disruption of natural ecosystems, such as habitat encroachment enabled by inadequate law enforcement and lax land-use regulations. For example, previous research on human-Andean-bear interactions in Colombia found evidence that governance-related issues, such as inadequate coordination and capacity within governmental agencies at regional levels were driving human-bear conflicts^43^. A study in Colombia reported evidence that weak governance of PAs negatively impacted law enforcement, increasing illegal activities like mining, coca plantations, and illegal timber and deforestation^44^.

In our analysis, we explored the impact of community participation (associated with the inclusivity domain of governance) on the risk of HWC^42^. Participation was the third most sensitive variable in reducing the risk of HWC. Participation is important for conservation: it enhances local community engagement, promotes a sense of ownership, and ultimately may lead to more sustainable conservation outcomes^45^. However, achieving robust participation is challenging due to communication gaps and a lack of clarity about how to be involved^46^. For instance, in Peru, ineffective participation was the primary cause of the failure of several conservation initiatives in the Amazon region^47^. Our work suggests that improving participation should improve governance, thus reducing the risk of HWC. Promoting effective engagement of local communities and stakeholders within PAs decision-making processes may be accomplished through activities such as consultations, workshops, and co-management. For instance, previous research demonstrated how integrating local knowledge in PAs stewardship enhanced decision-making accountability and strengthened the perceived legitimacy of PAs rules in Madagascar^46^.

Areas with higher habitat quality, as defined by forest integrity, had a lower risk of HWC. Our BBN model reveals that maximizing habitat quality results in a 26.4% reduction in the risk of HWC. Habitat quality is important for species fitness^48^ and connectivity^28^. For example, studies in Tanzania and Kenya have demonstrated how fencing reduced habitat connectivity and increased human-elephant conflicts^49^. Landscape disturbances like forest cover loss and degradation can force wildlife closer to human settlements, increasing interactions and competition for resources with humans^34^. In Nepal, for example, researchers linked human-leopard conflicts to a decrease in prey availability within fragmented landscapes and increased HWC^50^. Research on jaguar (*Panthera onca*) populations has demonstrated species preference for less disturbed sites, and most human-jaguar conflicts occur in ranching areas embedded in forested regions^51^. Our study highlights that prioritizing intact forest and landscape connectivity can enable species to access critical resources while minimizing negative encounters with humans.

Conservation planning can contribute to habitat improvement and regulate human activities within and around PAs. Treves et al. ^52^ highlighted the importance of a conservation planning process where experts, policymakers, and community members design and ensure effective and sustainable conservation actions on the landscape to mitigate HWC. Complementary strategies such as habitat restoration, invasive species management, responsible water management, and wildlife corridor creation further contribute to the comprehensive improvement of habitat quality while reducing the chances of conflict. Regular wildlife monitoring, community engagement, climate change adaptation, education initiatives, and collaborative partnerships are integral components of habitat quality.

There is substantial evidence of a negative relationship between low acceptance levels toward wildlife and a heightened risk of HWC occurrence^53,54^. Wildlife acceptance signifies a more active form of tolerance toward wildlife, which is crucial for fostering and sustaining conditions of coexistence^24^. Our research demonstrated that rates of low wildlife acceptance are 37.4% across the three countries. This is nearly 4.5 times higher than high wildlife acceptance (averaging 8.3% across the three countries). Maximizing people’s acceptance of wildlife results in high risk of HWC declining from 15.3% to 2.7%. This finding is supported by a study in Zambia that highlighted the importance of increasing acceptance and tolerance toward wildlife as an effective way to mitigate HWC^55^. Promoting the benefits of wildlife to communities is a well-established approach to increasing acceptance^24,55^. However, this can be challenging, particularly when the costs to livelihoods and safety compete with, or even outweigh the perceived benefits^56^. In practice, wildlife professionals often focus on the economic advantages of wildlife, assuming these are most tangible and easily understood by communities. However, this focus may overlook indirect benefits, such as personal fulfillment, cultural significance, and ecosystem services. To effectively foster wildlife acceptance, it is essential to consider both the specific benefits and challenges associated with each species. Because human-wildlife conflicts vary by location community context, tailored approaches that address local perspectives and experiences can lead to more effective and sustainable solutions.

Increasing the economic benefits of wildlife through tourism and recreational activities, coupled with the strategic distribution of these resources, can improve local livelihoods and foster greater wildlife acceptance. For instance, tourism centered around predators has been shown to positively influence people’s attitudes toward cougars^57^. Beyond economic incentives, promoting the cultural and spiritual significance of wildlife can strengthen community identity and values, leading to more positive attitudes. In Russia, for example, some ethnic groups revere bears and honor them in festivals as symbols of power and sacredness^58^. Additionally, recognizing wildlife as key providers of ecosystem services – such as pollination, seed dispersal, and pest control – highlights their essential role in maintaining ecological balance^59^. Integrating these economic, cultural, and ecological perspectives can create a more holistic approach to fostering coexistence between humans and wildlife.

Scalability is a key aspect in our approach; we emphasize that the BBN is flexible and adaptable across different scales and contexts. At the country level, our analysis identified governance as the most crucial variable, reducing variance by 26.2% in Colombia, 24.3% in Ecuador, and 27.9% in Peru. We opted to run separate country-specific models rather than a single integrated model because substantive differences in ecological conditions, governance structures, institutional capacity, monitoring systems, reporting practices, and data distributions across countries could obscure context-dependent dynamics relevant for interpretation and decision-making. Given the applied focus of this study, country-specific models allowed us to preserve context-specific interpretations and generate more actionable insights for national-level management and policy contexts. To apply the BBN at the local PA scale, we first verified whether the main model hypotheses held at that level, then refined the model using only responses from staff linked to the specific protected area. In Yasuni National Park, Ecuador, the risk of high HWC was 17%, with negative interaction with jaguars as the main concern. Sensitivity analysis showed habitat quality (18.9%) as the second most influential variable behind governance (24.1%). Maximizing HWC report frequency reduced the HWC risk from 17% to 4.5%, suggesting that, in this PA, focusing on wildlife acceptance may be more effective than reviewing governance. As shown, the BBN enables scaling from national to local levels and can assess HWC risks using species presence, altitudinal range, or ecosystem traits.

Based on our findings, managers may be interested in using BBN models in PAs to assess and estimate the risk of HWC for several reasons: (I) BBNs serve as a statistical and systematic tool, providing a structured and probabilistic approach to modeling uncertainties and relationships among diverse variables. This method helps reduce uncertainty in analyzing human-wildlife interactions and facilitates a more comprehensive understanding of HWC. By leveraging BBNs, stakeholders can improve collaboration and decision-making based on structured, data-driven insights; (II) Mitigating HWC risks not only strengthens the effectiveness of PAs but also contributes to species conservation. PAs have become a cornerstone for conservation, serving essential refuges for wildlife, particularly large mammals^60^. However, studies have shown that HWC often intensifies near PA borders due to human activities such as farming, ranching, and tourism in adjacent areas^56^.; (III) Using PA staff to monitor HWC can be a cost-effective strategy. Rangers with specialized training in community engagement play a critical role in managing relationships with local communities and assessing human-wildlife interactions. Integrating HWC monitoring into routine PA activities is a low cost for national or local institutions. Given that many park rangers originate from communities within or near PAs, their local knowledge and presence throughout the year can enhance the effectiveness of monitoring efforts.; (IV) BBN offer a rigorous framework for integrating ecological and social data, enabling truly interdisciplinary analyses; and (V) BBN models support analysis at multiple scales. At the local level, PA managers can use our model to understand how different variables, such as participation, law enforcement, and habitat quality, may affect HWC. At regional or national scales, our model can aid policymakers in identifying broader HWC trends and then develop strategies to mitigate conflicts at the landscape level. This multi-scale approach can improve policy formulation and target interventions to reduce HWC incidents.

The effectiveness of HWC interventions depends on the scale at which they are implemented. Our proposed model, designed for regional and broader applications, characterizes the dynamic nature of the interaction between people and wildlife and provides insights for conflict mitigation at the landscape scale. By supporting PA managers in decision-making, the model enhances wildlife acceptance and strengthens HWC monitoring efforts. Addressing HWC requires a holistic approach that accounts for its interconnected ecological, economic, social, and political dimensions. While the complexity of these conflicts may seem overwhelming, lessons from other global challenges such as climate change, overpopulation, and plastic pollution highlight the necessity of integrated, multi-faceted solutions. Our research underscores the need for adaptive, evidence-based strategies to foster coexistence between humans and wildlife while ensuring the long-term sustainability of both conservation efforts and local communities.

## Methods

### Study area

**Fig. 6:**
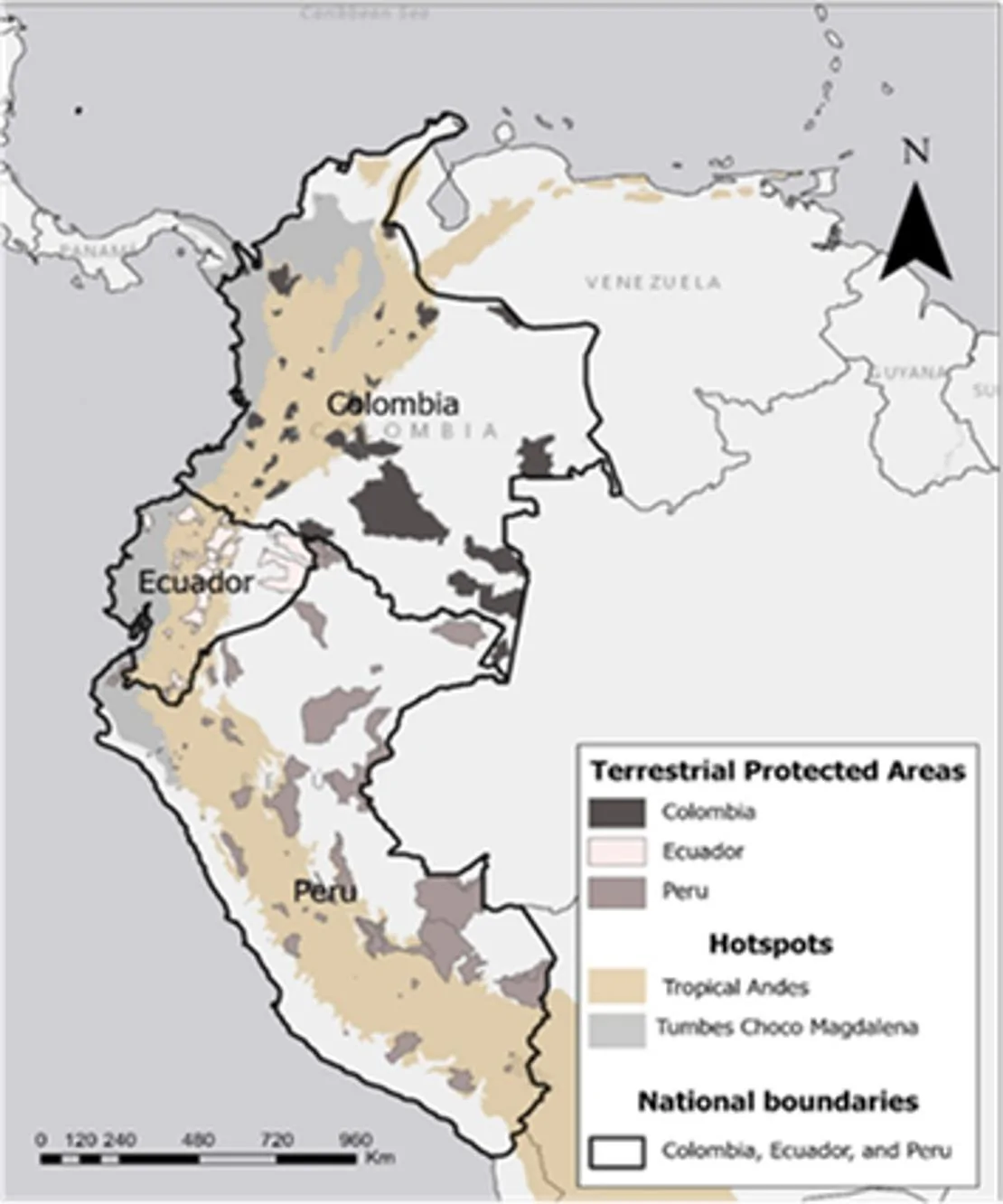
Study Site. Study site. Protected Areas of Colombia, Ecuador, and Peru, including the Tropical Andes and Tumbes–Chocó–Magdalena Biodiversity Hotspots. The map was created by the authors using ArcGIS Pro 3.5.2.

The study area included 135 protected areas (PAs) in three countries: Colombia (*n* = 42), Ecuador, (*n* = 35), and Peru (*n* = 58) (See Fig. 6). The establishment and execution of PAs have been a priority for regional and national governments to preserve natural ecosystems. Presently, the coverage of PAs is 16.4% in Colombia, 23.4% in Ecuador, and 22.5% in Peru^61^.

The study region overlaps with two critical biodiversity hotspots, the Tropical Andes Hotspot, which is the most biodiverse area in the world by the number of plants and mammal species^62^, and the Tumbes Choco Magdalena that comprises diverse ecosystem types (coastal and marine, tropical dry forests, and wetlands ecosystems) and cultural diversity stemming from several ethnic groups that inhabit the region^63^. Colombia, Ecuador, and Peru share similar climates and topography and are part of the Tropical Andes subregion that supports an estimated 45,000 plants and 3400 vertebrate species (excluding fishes)^64^.

Despite the biological richness of this region, it faces severe social and environmental threats like rapid economic growth and inequity, agricultural intensification, infrastructure development, high population density, and a high dependence on natural resources, among other pressures^65^. Most of the conservation priority areas in Colombia and Ecuador are located near urban areas (König et al., 2020). In Latin America and the Caribbean (LAC) region, over 25% of the urban population experiences extreme poverty, with the wealthiest 20% earning 20 times more than the least affluent 20% ^65^. There are observed correlations between biodiversity decline and poverty in tropical regions.

### Survey development

We designed a PA manager survey to provide them with information they can use to design better strategies to mitigate the costs of human-wildlife conflict (HWC) based on the type of conflicts and species involved. The goal was to help decision-makers prioritize resources and define strategies to promote coexistence in the PAs.

As a part of survey development, we conducted three virtual meetings in 2020 with PA managers and wildlife specialists from the Ministry of Environment, Water and Ecological Transition of Ecuador (MAATE). We selected a candidate set of governance and wildlife acceptance variables from literature and validated/refined them with PA managers and wildlife specialists. These variables were then used to develop the model variables and survey questions, with the full justification and references provided in the Supplementary Materials. The survey was designed to compile information about HWC within the last three years within PAs, plus a buffer zone of 3 km around the PA boundary. We included questions related to the frequency of negative encounters with wildlife, the species involved, and the type of conflict (Table 2). We also asked participants to rank the top three species most usually involved in HWC within the PAs. The survey included 34 questions divided into four sections: consent and participant demographic information, HWC assessment, and a section focusing on our Bayesian Belief Network (BBN) model that included aspects related to governance (law enforcement, law acceptance, participation, education) and wildlife acceptance (tolerance, attitudes, human-wildlife conflict report frequency, distance of conflict events to human settlements). We included multiple choice, ranking, mapping, and Likert-scale survey questions. In Colombia and Peru, we used the survey developed for Ecuador but modified the species list, so it was representative of species in those countries.

**Table 2:** Variables included in the Bayesian Belief Network model to predict the risk of future human-wildlife conflict. The survey information was gathered from personnel associated with protected areas (PA), including protected area managers, park rangers, and specialists across Colombia, Ecuador, and Peru.

Variable	Definition	Questions	States	Source
Attitude	Attitude is a summary evaluation of an object shaped by personal experience, beliefs, and emotions. It could define a person’s predisposition toward or against something.	When you interact with local communities about wildlife issues, what is their reaction towards wildlife?	Negative Neutral Positive	Survey
Education	Local communities’ education received about the existence of wildlife, the services they provide, and the rules of the protected area.	How often have you organized events to share information with local communities about the environmental services provided by forests and wildlife to people and explain the protected area rules?	Weekly Monthly Every six months Annually Never	Survey
Wild_crim_freq	Frequency in which PA staff receive complaints or alerts from community members about wildlife crimes such as incidents of illegal hunting or fishing within the protected area.	How often did you receive complaints or were you alerted by members of the community about incidents of illegal hunting or fishing carried out by other members of the community within the protected area zone? (+ 3 km buffer zone)?	Weekly Monthly Every six months Annually Never	Survey
Law acceptance	Level of acknowledging and following the rules and law. It is about obeying and recognizing what is legally required or expected within a PA.	How often have people from the local communities within the protected area (+ 3 km buffer zone) protested or made claims regarding a protected area policy or decision related to using forests and wildlife within the protected area? For example, protests against prohibitions of logging, hunting, and road building, among others)	Weekly Monthly Every six months Annually Never	Survey
Law enforcement	The capacity of the protected area administrators to discover, deter, or punish people who violate the rules and norms of the protected area.	How difficult is it to enforce the protected area rules and get people to comply?	It is not difficult at all Slightly difficult Very difficult	Survey
Participation	Engagement of local communities and stakeholders in the decision-making processes and activities of the protected area.	How often have people from the local communities within the protected area (+ 3 km buffer zone) contacted you to influence or change any decision regarding the use of forests and wildlife within the protected area? Contact forms include letters, emails, calls, conversations, public audience, etc.	Weekly Monthly Every six months Annually Never	Survey
Distance	Distance of conflicts to human settlements. Events far from human settlements could be perceived as less important than events close to communities.	Based on your experience working in the protected area, at what distance from human settlements do human-wildlife conflicts usually occur?	Near (< 500 m) Far (> 500 m)	Survey
Rangers	A protected area with an adequate number of rangers is more likely able to accomplish its conservation goals.	In the last three years, how do you think the number of park rangers working in the protected area has changed within the protected natural area and its 3 km buffer zone?	Decrease Same Increase	Survey
HWC_report_freq	People report human-wildlife conflicts to local authorities, avoiding solving the problem independently This node was conceived as a proxy for social norms.	How often have you received complaints about human-wildlife conflicts (attacks on people, crops, or livestock)?	Weekly Monthly Every six months Annually Never	Survey
Tolerance	Tolerance is the ability or willingness to accept something, in this case, the presence of wildlife species that cause damage.	People tolerate most damage (to people or property) from wildlife.	Strongly disagree Disagree Neutral Agree Strongly agree	Survey
Tourism	Visitation of the protected area by tourists.	How has the number of tourists visiting the protected area changed in the last three years?	Decreased Same Increased	Survey
Governance	The effectiveness of protected area administrators in achieving conservation and their ability to enforce laws and ensure stakeholders comply with norms and regulations.	This variable is defined by a deterministic approach using Law enforcement and Participation.	Low Medium High	Expert Opinion
Habitat Quality	Index of forest integrity as determined by degree of anthropogenic modification	We used the forest landscape integrity Index to define the quality of habitat in each protected area + 3 km edge zone.	Low Medium High	Index
Wildlife acceptance	Level of the willingness of local communities to coexist with wildlife.	This variable is defined by a deterministic approach using Tolerance and human-wildlife conflict report frequency.	Low Medium High	Expert Opinion
Risk_HWC	Output variable that estimates the likelihood of the future risk of human-wildlife conflict occurrence.	This variable is defined by a deterministic approach using Governance, Habitat Quality, and Wildlife acceptance.	Low Medium High	Expert Opinion

We conducted virtual meetings with representatives from the National Natural Parks of Colombia (NNPC) and the National Service of Natural Areas Protected by the State (SERNANP), where we presented our BBN model network structure and explained the cause-effect relationship among the model variables. We received feedback from the participants on survey design: in response, we clarified the common names of species and included other species of interest. We jointly defined the final variables and discussed rules to define the contingency tables to estimate governance, wildlife acceptance, and the risk of HWC occurrence. In September 2020, we conducted three virtual meetings one for each geographical region (Coast, Andean, and the Amazon) with representatives from Ecuador’s PAs, totaling 91 participants. In June 2021, two regional workshops were organized for Peru (one included the PAs from the coast and Andean regions, and other for PAs from the Amazon region) involving 56 PA’s representatives. Subsequently, a national virtual meeting for Colombia took place in June 2022 with 28 representatives. During these sessions, we engaged PAs’ staff in reviewing the conceptual model network and variables. Additionally, an online pre-survey was administered to familiarize participants with survey questions.

Species lists were developed in coordination with PA managers and wildlife specialists. They encompass a predetermined set of the most widely recognized species involved in HWC, such as large carnivores, birds of prey, and species known for raiding crops. Additionally, we included generalist species such as snakes and bats, which people often stigmatize as harmful. We included 41 species of interest in the three countries (Supplementary Table 4): Colombia (*n* = 26), Ecuador (*n* = 23), and Peru (*n* = 37).

To identify types of HWC, we asked participants about events in the last three years within the protected area zone, including the 3 km buffer. We used six categories for conflicts in terrestrial protected areas: damage to wildlife, crop raiding, livestock attacks, attacks on domestic animals other than livestock, disease transmission, and threats to people.

### Bayesian Belief Network

We used manual construction to develop the BBN^66,67^, relying on domain knowledge and expert validation with PA managers to establish the structure of our BBN model. This entails identifying crucial variables, particularly target variables (outputs) and observation variables (inputs). The initial stage involves selecting important variables identified as important in HWC literature, to draft an initial model structure. The authors examined each dependent and independent relationship within our initial model. This involved a thorough analysis and validation process, where hypotheses regarding cause-and-effect relationships were carefully scrutinized based on HWC literature.

The next step included validation of our model and contingency tables with the PAs’ personnel, including PA managers, park rangers, and PAs’ specialists from Colombia, Ecuador, and Peru via online work sessions.

Eleven variables were included in the survey. To define variables such as governance, acceptance of wildlife, and risk of HWC, we used expert opinion to define a scenario planning approach, where we identified the best-worst case approach and considered constrained scenarios, as a means to explore different future possibilities and reduce uncertainty^68^. We used constrained scenarios to develop contingency tables that allowed us to project possible scenarios (e.g., low, medium, or high levels of governance). These scenarios were validated by PA managers and specialists during a workshop and virtual calls with representatives of MAATE and SERNANP, respectively. The scenario planning approach facilitates decision-makers’ insights into uncertainties, enabling the creation of strategies that navigate constraints while allowing them to better adapt to various conditions to identify alternatives for HWC. We ran our model separately for each country due to social, ecological, economic, political, and geographical differences among the three countries. We used the risk of HWC as our outcome variable based on fourteen variables representing three categories: governance, habitat quality, and wildlife acceptance. In our model, we represent causality using nodes and arcs. The arcs represent the causal condition dependency between two nodes (See Table.S2).

**Fig. 7:**
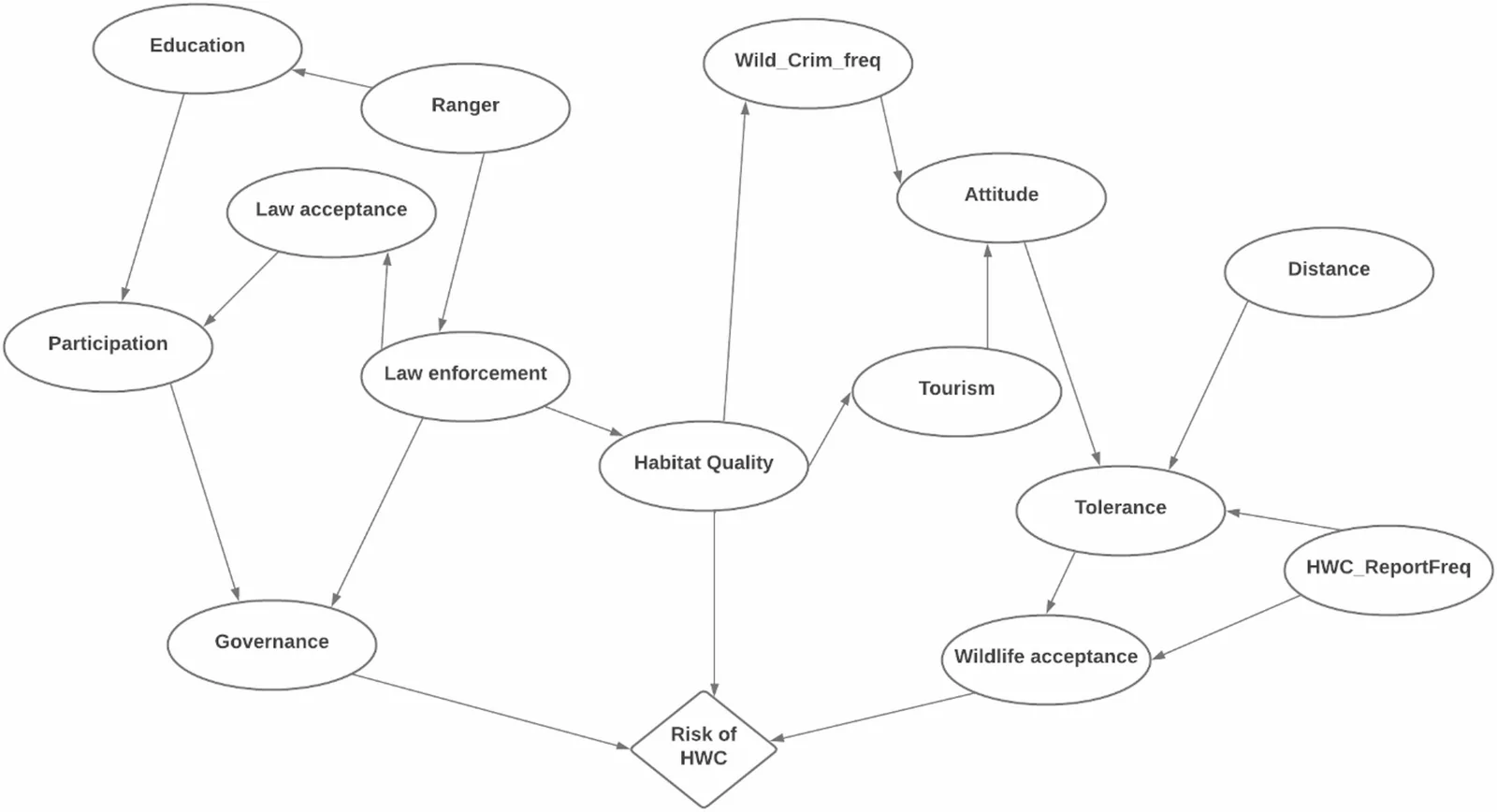
Model Network Structure for a Bayesian Belief Network. Structural Model to estimate the risk of future human-wildlife conflict (HWC) occurrence in terrestrial protected areas of Colombia, Ecuador, and Peru.

We used 15 variables in our BBN. Figure 7 shows our network structure for terrestrial PAs.

### Governance

The deterministic rules used to define the conditional probability table to estimate governance are described in Table 3. We applied a deterministic rule to classify governance levels. The worst-case scenario, defined by low participation and difficulties in law enforcement, was categorized as weak governance. The best-case scenario, combining high participation with no enforcement difficulties, was classified as strong governance. All other combinations were assigned to the medium governance category. This rule-based approach simplifies decision-making perspectives by offering a clear, transparent framework for categorizing governance conditions.

**Table 3:** Contingency table to estimate the level of governance included in the Bayesian Belief Network model to predict the risk of future human-wildlife conflict within protected areas (PA) of Colombia, Ecuador, and Peru.

Participation					
Variable States	Weekly	Monthly	Every six months	Annually	Never
Law enforcement					
Not difficult	Strong	Strong	Medium	Medium	Medium
Slightly difficult	Strong	Medium	Medium	Medium	Weak
Very Difficult	Medium	Medium	Medium	Weak	Weak

We define “Participation” as the degree to which people are informed or involved in PA decisions. For “Law enforcement” we refer to the capacity of PA to apply their rules. " Variable states” refer to the different options or categories that a variable can have. Variable states in Bayesian networks are essential for dynamic probability calculations, enabling the network to update and adjust predictions based on the current conditions of variables and represent complex relationships among factors in a system.

### Habitat quality

We measured habitat quality using the Forest Landscape Integrity Index (FLII) (Grantham et al., 2020a). The FLII is an annual global index of forest conditions measured by the degree of anthropogenic modification to measure global landscape integrity (https://www.forestintegrity.com/). The index uses information related to (i) forest extent that includes vegetation > 5 m in height in the year 2019; (ii) observed human pressure that can be mapped as human infrastructure present in the landscape; (iii) inferred human pressure that cannot be mapped that occurs in association with observed human pressure; a good example can be the collection of forest materials, crops, hunting or wildfires; and (iv) Loss of forest connectivity^69^.

The Forest Landscape Integrity Index is calculated at the pixel level (300 m) using the formula:

$$\:{FLII}_{i}=[10/3]\mathrm{*}(3\:-\mathrm{m}\mathrm{i}\mathrm{n}\left(3,\left[{P}_{i}+{\mathrm{Q}}_{i}+{\mathrm{L}\mathrm{F}\mathrm{C}}_{i}\right]\right))$$

Where: $\:{P}_{i}$= Observed human pressure refers to pressure that can be directly mapped like agriculture, tree cover loss, or infrastructure. $\:{\mathrm{Q}}_{i}$= Inferred human pressure refers to the edge effect of observed human pressures. $\:{\mathrm{L}\mathrm{F}\mathrm{C}}_{i}$= Loss of forest connectivity refers to changes in landscape connectivity.

The index ranges from 0 to 10, and values are grouped into three categories:

- High Forest Integrity (scores ≥ 9.6): comprised entirely or almost entirely of native species and natural edges of unmodified naturally regenerated forest ecosystems or areas with little human use.
- Medium Forest Integrity (scores > 6 but < 9.6): areas dominated by native species but substantially modified by human interventions, including minimal fragmentation, selective logging, and moderate changes due to fires and hydrological regimes.
- Low Forest Integrity (scores ≤ 6): includes diverse ranges of intensive human modifications such as fragmentation, changes in species composition, and agroforestry, among others.

### Wildlife acceptance

We used wildlife acceptance as a proxy for tolerance toward wildlife. Acceptance and tolerance have been used similarly in the literature on HWC to refer to passive coexistence with wildlife that involves the lack of negative behaviors toward species^70,71^. In our model, wildlife acceptance is a construct informed by the frequency with which human–wildlife conflict is reported (HWC_report_freq), which can shape people’s attitudes toward wildlife alongside the perceived benefits from tourist activities. In addition to attitude, we used the distance between human settlements and where HWC usually occurs, together with wildlife crime frequency (Wild_crim_freq), to define tolerance. The distance variable is not intended to measure the severity of loss, but rather the perceived salience and psychological proximity of conflict. Conflicts occurring farther from settlement cores may be perceived as less immediate or less threatening, even when they still generate livelihood losses. At the same time, because agricultural and pastoral land use often occurs beyond settlement cores, reported distance may partly reflect land-use configurations (e.g., crops or livestock located > 500 m from homes), which introduces interpretive ambiguity; therefore, this variable should be interpreted as perceived proximity rather than objective harm. Wildlife crime frequency was included as a proxy for community responses to harmful activities toward wildlife, under the assumption that more frequent reporting of illegal hunting or fishing may reflect lower tolerance for wildlife crime, greater awareness, and stronger pro-conservation attitudes. Wildlife acceptance is then classified using a deterministic function of wildlife crime frequency and tolerance (Table 4).

**Table 4:** Contingency table to describe the level of wildlife acceptance included in the Bayesian Belief Network model to predict the risk of future human-wildlife conflict within protected areas of Colombia, Ecuador, and Peru.

Human-wildlife conflict report frequency					
Variable states	Weekly	Monthly	Every six months	Annually	Never
Tolerance					
Strongly disagree	Medium	Medium	Medium	Low	Low
Disagree	Medium	Medium	Medium	Low	Low
Neutral	High	High	Medium	Low	Low
Agree	High	High	Medium	Medium	Medium
Strongly agree	High	High	Medium	Medium	Medium

We refer to “variable states” as the different categories or levels that a variable can take. Tolerance is defined as people’s willingness to accept the presence of wildlife. The variable Human-wildlife Conflict Report Frequency captures the extent to which individuals report human–wildlife conflicts to local authorities rather than addressing them independently. While not a direct measure of social norms, this variable may partly reflect shared expectations about appropriate responses to conflict situations.

### Risk of human-wildlife conflict

The risk of future HWC or risk of HWC (Risk_HWC) was established using a deterministic function as a combination of the interaction of governance, acceptance toward wildlife, and habitat quality (Table 5).

**Table 5:** The contingency table describes the future level of risk of human-wildlife conflict (HWC) and the “Risk of HWC” within the Bayesian Belief Network model for protected areas of Colombia, Ecuador, and Peru. Wildlife acceptance refers to the level of tolerance toward wildlife. Habitat quality describes how well the landscape supports wildlife survival, which tends to decrease with increasing disturbance. Governance reflects the protected area’s capacity to apply laws and policies that enable collective decision-making.

Wildlife Acceptance	Habitat Quality	Governance	Risk of HWC
Low	Low	Low	High
Low	Low	Medium	High
Low	Low	High	High
Low	Medium	Low	High
Low	Medium	Medium	Medium
Low	Medium	High	Medium
Low	High	Low	High
Low	High	Medium	Medium
Low	High	High	Low
Medium	Low	Low	High
Medium	Low	Medium	Medium
Medium	Low	High	Medium
Medium	Medium	Low	Medium
Medium	Medium	Medium	Medium
Medium	Medium	High	Medium
Medium	High	Low	Medium
Medium	High	Medium	Medium
Medium	High	High	Low
High	Low	Low	High
High	Low	Medium	Medium
High	Low	High	Low
High	Medium	Low	Medium
High	Medium	Medium	Medium
High	Medium	High	Low
High	High	Low	Low
High	High	Medium	Low
High	High	High	Low

#### Data collection

The online questionnaire was delivered using Qualtrics software. It was written in Spanish and available for completion in different formats, including desktop computers, smartphones, and tablets. Each country’s respective PAs’ national authority emailed the survey link to protected area managers. To increase the response rate, we provided information support to responders on completing the survey by implementing virtual office hours, WhatsApp group texting, and assistance by email.

The survey was addressed to every person > 18 years old who worked in a protected area in any of the three countries, including managers, park rangers, and wildlife specialists. Respondents answered as individuals rather than pooling their responses by PA. The survey was conducted in Ecuador between 30 September and 15 November 2020, in Peru between 11 June and 18 October 2021, and in Colombia between 7 July and 20 October 2021. The survey duration was longer in Peru and Colombia because PAs are often located in remote areas, and their staff faced challenges in accessing the iinternet. Finally, we generated a WhatsApp texting group for each country that was available when the survey was opened for all the survey participants. We used the WhatsApp texting group to share a video tutorial explaining how to use Qualtrics to complete the survey.

This study was approved by the Cornell Institutional Review Board (Approval No. IRB0001927). All methods were carried out in accordance with relevant guidelines and regulations. Informed consent was obtained from all participants prior to their participation in the online survey, and participants were informed about the purpose of the study, data confidentiality, and their right to withdraw at any time.

## Supplementary Information

Below is the link to the electronic supplementary material.

Supplementary Material 1

## Data Availability

Data have limited availability owing to sensitivity concerns. Contact Dr. Santiago Garcia for more information.
