# Supplementary material for: Bayesian belief network model to predict human-wildlife conflict in protected areas
Source: Sci Rep. 2026 Jun 19;16:26502. doi: 10.1038/s41598-026-56081-9 (PMC13503659; doi:10.1038/s41598-026-56081-9)
Supplement: Supplementary file 1 [file 41598_2026_56081_MOESM1_ESM.docx]

**Supplementary Information**

# Bayesian Belief Network Model to Predict Human-Wildlife Conflict in Protected Areas.

**Santiago García-Lloré ^1, +, *^, Richard C. Stedman ^2, +, *^, and Angela K. Fuller ^3 +, *^**

^1^New York Cooperative Fish and Wildlife Research Unit, Ashley School of Global Development and the Environment, Cornell University; Ithaca, 14853 New York, USA.

^2^ Ashley School of Global Development and the Environment, , Cornell University; Ithaca, 14853 New York, USA.

^3^U.S. Geological Survey, New York Cooperative Fish and Wildlife Research Unit, Ashley School of Global Development and the Environment, Cornell University; Ithaca, 14853 New York, USA.

*Corresponding: sag337@cornell.edu (S.G.); angela.fuller@cornell.edu (A.F.); rcs6@cornell.edu (R.S.)

Any use of trade, product, or firm names is for descriptive purposes only and does not imply endorsement by the U.S. Government.

**Supplementary Tables**

Between September 2020 and November 2021, we received 1417 responses from protected areas (PAs) employees (managers, park rangers, and other staff members);137 responses were excluded following review, including 96 that were categorized as NA due to not completing minimal information in the survey like PA name, and 41 that were removed because they represented duplicate responses .Although we included 194 PAs in the survey, we only received responses from 176. We did not receive responses from four PAs in Colombia, nine PAs in Ecuador, and nine PAs in Peru.

After reviewing and cleaning the survey data, 1,280 valid responses were included from 176 PAs of Colombia, Ecuador, and Peru (Supplementary Table 1).

***Supplementary table 1.*** *Number of completed survey responses received about human-wildlife conflicts within protected areas by type of protected area: marine protected area (MPA), terrestrial protected area (TPA) and the total of both categories of protected areas (PA) in Colombia, Ecuador, and Peru.*

|  | **Marine Protected Areas** | | **Terrestrial Protected Areas** | | **Total** | |
| --- | --- | --- | --- | --- | --- | --- |
| **Countries** | **MPA** | **Responses** | **TPA** | **Responses** | **PA** | **Responses** |
| Colombia | 16 | 39 | 42 | 175 | 58 | 214 |
| Ecuador | 17 | 171 | 35 | 481 | 52 | 652 |
| Peru | 8 | 59 | 58 | 355 | 66 | 414 |
| **Total** | 41 | 269 | 135 | 1011 | 176 | 1280 |

Respondents included 95 PA managers, 293 PA specialists, 893 park rangers, 9 PA assistants, and 61 PA staff in “other” categories among the three countries (Supplementary Table 2).

***Supplementary table 2.*** *Roles of staff wi*th*in the survey about human-wildlife conflicts in Protected Areas of Colombia, Ecuador, and Peru. The “Others” categories include roles specific to each country, such as Environmental Educator, Natural Resources Analyst, Forestry Extension Agent, Zone Coordinator, Community Reservist, Indigenous Operator, and Tourism Specialist.*

| **Role within the Protected Area** | **Colombia** | **Ecuador** | **Peru** | **Total** |
| --- | --- | --- | --- | --- |
| Protected Area Manager | 11 | 47 | 37 | 95 |
| Protected Area Specialist | 129 | 57 | 107 | 293 |
| Park Ranger | 61 | 523 | 238 | 823 |
| Protected Area Assistant | 5 | NA | 4 | 9 |
| Others | 8 | 25 | 28 | 61 |
| Total | 214 | 652 | 414 | 1280 |

Spearman rank correlations were conducted because both HWC_report_freq and Wild_crim_freq are ordinal variables. Results showed positive and statistically significant associations across the full sample and within all countries (**Supplementary table 3**). Although the variables were related, correlation values remained within the low-to-moderate range, suggesting some shared information but also meaningful conceptual non-overlap. Therefore, both variables were retained in the Bayesian Belief Network models.

| **Supplementary table 3.** Spearman rank correlations between human–wildlife conflict report frequency (HWC_report_freq) and wildlife crime frequency (Wild_crim_freq) across the full sample and by country. | | | |
| --- | --- | --- | --- |
| **Dataset** | **Spearman’s ρ** | **p-value** | **n** |
| Full sample | 0.361 | 1.78 × 10⁻¹⁵ | 455 |
| Colombia | 0.39 | 0.000121 | 92 |
| Ecuador | 0.409 | 1.11 × 10⁻¹¹ | 254 |
| Peru | 0.255 | 0.00752 | 109 |
| Missing values coded as "*" were excluded from the analysis. Positive and statistically significant correlations were observed across the full sample and within all countries. Correlation values were low to moderate, indicating partial overlap but not severe collinearity or complete redundancy between variables. | | | |

Species lists were developed in coordination with PAs managers and wildlife specialists. They encompass a predetermined set of the most widely recognized species involved in HWC, such as large carnivores, birds of prey, and species known for raiding crops. Additionally, we included generalist species such as snakes and bats, which people often stigmatize as harmful. We included 41 species of interest in the three countries (Supplementary Table 4): Colombia (n = 26), Ecuador (n = 23), and Peru (n = 37).

Supplementary Table 4. Species identified by protected area managers in Colombia, Ecuador, and Peru have the potential for human-wildlife conflict in terrestrial or coastal/marine areas. Some species are cataloged only by family (e.g., bats, snakes, parrots, dolphins, sea lions, and deer).

| **Family name** | **Scientific Name** | **Common Name** | **Terrestrial** | **Coastal** | **Colombia** | **Ecuador** | **Peru** |
| --- | --- | --- | --- | --- | --- | --- | --- |
|  |  |  |  | **& Marine** |  |  |  |
| Cervidae |  | Deer | x |  | x |  | x |
| Cuniculidae | *Cuniculus paca* | Lowland paca | x |  | x |  | x |
| Mephitidae |  | Skunk | x |  |  |  | x |
| Pelecanidae | *Pelecanus occidentalis* | Brown pelican |  | x | x | x | x |
| Alligatoridae |  | Caiman | x |  |  |  | x |
| Balaenopteridae | *Megaptera novaeangliae* | Humpback.whale |  | x | x | x | x |
| Bovidae | *Capra hircus* | Goat |  | x |  |  | x |
| Camelidae | *Vicugna vicugna* | Vicuna | x |  |  | x | x |
| Canidae | *Canis familiaris* | Domestic dog | x | x | x | x | x |
| Canidae | *Lycalopex culpaeus* | Culpeo | x | x | x | x | x |
| Canidae | *Lycalopex sechurae* | Sechuran fox |  | x |  |  | x |
| Cathartidae | *Vultur gryphus* | Andean condor | x | x | x | x | X |
| Cheloniidae | *Eretmochelys imbricata* | Hawksbill sea turtle |  | x | x | x | X |
| Cheloniidae | *Chelonia mydas* | Green sea turtle |  | x | x | x | X |
| Crocodylidae | *Crocodylus acutus* | Crocodile |  | x | x | x | X |
| Delphinidae |  | Dolphin |  | x | x | x | X |
| Dermochelyidae | *Dermochelys coriacea* | Leatherback sea turtle |  | x | x | x | X |
| Didelphidae | *Didelphis sp* | Opossum | x |  |  |  | X |
| Equidae | *Equus asinus* | Donkey |  | x |  |  | X |
| Felidae | *Panthera onca* | Jaguar | x | x | x | x | X |
| Felidae | *Leopardus Sp.* | Ocelots/Oncilla | x |  | x | x | X |
| Felidae | *Puma concolor* | Cougar | x | x | x | x | X |
| Laridae |  | Seagull |  | x | x | x | X |
| Olive ridley sea turtle | *Lepidochelys olivacea* | Olive ridley sea turtle |  | x | x | x | X |
| Otariidae |  | Sealion |  | x | x | x | X |
| Psittacidae | *Ara sp* | Macaw | x |  |  |  | X |
| Psittacidae |  | Parrot | x |  |  |  | x |
| Spheniscidae |  | Penguin |  | x |  |  | x |
| Tapiridae | *Tapirus terrestris* | Amazonian.Tapir | x |  |  | x | x |
| Tapiridae | *Tapirus pinchaque* | Mountaintapir | x |  | x | x | x |
| Tapiridae | *Tapirus terrestris* | Lowland.Tapir | x |  | x |  |  |
| Tayassuidae | *Tayassu pecari* | White_lipped.peccary | x |  | x | x | x |
| Tayassuidae | *Pecari tajacu* | Collared peccary | x |  | x | x | x |
| Ursidae | *Tremarctos ornatus* | Andean bear | x |  | x | x | x |
|  |  | Bat | x | x | x | x | x |
|  |  | Snake | x | x | x |  | x |
| Canidae | *Canis latrans* | Coyote | x |  | x |  |  |
| Canidae | *Cerdocyon thous* | Crab-eating fox | x |  |  |  |  |
| Mustelidae | *Neogale frenata* | Long-tailed weasel | x |  |  |  | x |
| Procyonidae | *Procyon cancrivorus* | Crab-eating raccoon |  | x |  |  | x |
